# Coupling of CSF and sagittal sinus pressure in adult patients with pseudotumour cerebri

**DOI:** 10.1007/s00701-019-04095-w

**Published:** 2019-12-12

**Authors:** Afroditi-Despina Lalou, Marek Czosnyka, Zofia H. Czosnyka, Deepa Krishnakumar, John D. Pickard, Nick J. Higgins

**Affiliations:** 1 Division of Neurosurgery, Department of Clinical Neurosciences, Cambridge University Hospital, Cambridge, UK; 2 Department of Paediatric Neurology, Cambridge University Hospital, Cambridge, UK; 3 Department of Radiology, Addenbrooke’s Hospital, Cambridge, UK

**Keywords:** CSF pressure, Intracranial pressure, Idiopathic intracranial hypertension, Pseudotumour cerebri, Sagittal sinus pressure, CSF outflow resistance

## Abstract

**Objective:**

Pseudotumour cerebri syndrome (PTCS including idiopathic intracranial hypertension) is characterised by the symptoms and signs of raised cerebrospinal fluid pressure (CSFp) in the absence of ventricular dilatation or an intracranial mass lesion. Its aetiology is unknown in the majority of cases but there is much evidence for impaired CSF absorption. Traditionally, sagittal sinus pressure has been considered to be independent of CSF pressure in adults. However, the discovery of stenoses of intracranial venous sinuses and introduction of venous sinus stenting has highlighted the importance of the venous drainage in PTCS. In this study, we have explored the relationship between CSFp and SSp before and during a CSF infusion test and during CSF drainage.

**Materials and methods:**

Ten patients (9 females:1 male) with PTCS underwent infusion studies in parallel with direct retrograde cerebral venography. Both SSp and CSFp were recorded at a baseline and during CSFp elevation in a course of a CSF infusion test. The drainage of CSF after the CSF infusion was performed in 7 patients. In 5 cases, jugular venous pressure was also measured.

**Results:**

CSFp and SSp including their amplitudes correlated significantly and strongly both at baseline (*R* = 0.96; *p* = 0.001) and during infusion (*R* = 0.92; *p* = 0.0026). During drainage, this correlation was maintained until SSp reached a stable value, whereas CSFp continued to decrease.

**Conclusions:**

In this series of ten patients with PTCS, CSFp and SSp were coupled, both at baseline and during infusion. The implications of such coupling for the calculation of CSF outflow resistance are discussed.

## Introduction

Pseudotumour cerebri syndrome (PTCS) is characterised by the symptoms and signs of raised cerebrospinal fluid pressure (CSFp) in the absence of ventricular dilatation or an intracranial mass lesion and often without a known aetiology [10, 15, 16, 27, 28]. It mostly affects women of reproductive age with an increased body mass index (idiopathic intracranial hypertension, IIH) but less frequently can involve paediatric patients and patients regardless of their biological sex [15–17]. MRI reveals dilatation of the cortical subarachnoid space and optic nerve sheaths with compression of the pituitary gland (empty sella) [9].

Potential underlying mechanisms for PTCS include impaired CSF drainage and raised intracranial venous sinus pressure [12, 13, 25, 26]. There is little evidence for CSF hypersecretion or cerebral oedema. There is debate as to whether changes in CSF outflow resistance are sufficient per se to explain the raised CSF pressure in all patients [23]. Importantly, impaired CSF absorption may be caused by raised intracranial venous sinus pressure. Early studies of PTCS demonstrated abnormalities of intracranial venous drainage but later MR venography studies were misinterpreted as flow artefacts [1, 11, 17, 24, 31]. However, retrograde cerebral venography and CT venography demonstrated stenoses of the transverse sinuses in many patients with PTCS [12, 13, 25]. Commonly, patients with PTCS that is refractory to medical management have been offered surgery including various CSF diversion procedures and bariatric surgery [10, 31]. The demonstration of venous sinus stenoses with significant pressure gradients led to the introduction of venous sinus stenting in 2002 [13, 18].

Many of these stenoses were not fixed but resolved, at least in part, with drainage of CSF. Such reversible stenoses contradict the traditional view that the intracranial venous sinuses are largely incompressible in adults with sagittal sinus pressure being independent of CSF pressure [24]. Furthermore, these stenoses might create a positive feedback loop between increased CSFp and SSp in patients with PTCS with secondary impairment of CSF absorption [28]. There is increasing debate in developing the evidence behind shunting, therefore decreasing CSFp, and stenting, therefore decreasing venous pressure and ‘normalising’ cerebral venous anatomy and compliance, in PTCS. On the other hand, there is very little evidence on the pathophysiology of the increase of the two pressures, how they interact, and why each of the selected treatments could be effective or not.

In this study, we have explored the degree of coupling between CSFp and SSp waveforms in adults suffering from PTCS by using lumbar CSF infusion studies to measure CSFp with simultaneous direct measurements of their SSp at baseline, during infusion, and during/after CSF drainage. In addition, we have examined the implications of such coupling for the calculation of CSF outflow resistance when using Davson’s equation: CSFp = Rout × *I*_f_ + SSp, where Rout is the resistance to CSF outflow and *I*_f_ is the CSF formation rate [6].

A preliminary account of this work has been published [28].

## Patients and methods

Between 2004 and 2006, we investigated 10 patients (9F:1M) with the clinical features of PTCS who fulfilled the modified Dandy criteria (signs and symptoms of raised ICP, no localising neurological signs, normal neuroimaging apart from MR venography, raised CSF pressure (> 20 mmHg [8, 10, 15, , 27]), and normal CSF constituents). Their mean age was 41 years (range 22–55). All the patients had both headaches and papilloedema.

As part of their clinical investigation, they underwent two procedures: constant-rate lumbar CSF infusion studies, to assess the CSFp and CSF dynamics, and direct retrograde cerebral venography (DRCV) whereby a catheter was placed within the sagittal sinus under fluoroscopic guidance, in order to assess the significance of the stenosis.

Patients undergo either or both procedures in our hospital routinely as part of their PTCS investigations, in order to establish a diagnosis and plan treatment. Both DRCV and lumbar infusions have been used safely in our centre as well as other centres internationally [2, 4, 18].

### Lumbar infusion studies [2, 4, 7]

Access was gained via lumbar puncture using two 21-gauge Quincke needles at the intervertebral space L4-L5 using lidocaine local anaesthesia, with the patient lying on their side. A strict aseptic technique was used to keep all the pre-filled tubing and the transducer sterile. The skin was carefully prepared with antiseptic solution. Connection of a standard, disposable fluid-filled pressure transducer (Edwards Lifesciences^TM^ manometry lines, length 180 cm and inner diameter 1.2 mm), and pressure amplifier (Spiegelberg or Philips) to the LP needle allowed for pressure recording at a frequency of 30–100 Hz, with following processing by ICM+ (University of Cambridge Enterprise Ltd.) [4, 30].

Once a satisfactory CSFp pulse waveform had been achieved, baseline measurements were taken for 10 min, followed by infusion of Hartmann’s solution at 1.5 ml/min or 1.0 ml/min if the baseline CSF was ≥ 15 mmHg until the ICP had plateaued for 5–10 min. The protocol included a safety measure that required the infusion to stop if the mean ICP increased to 40 mmHg or above. This did not occur in any of these patients. The total duration of the infusion tests was 30 to 45 min. After the end of the infusion test, pressure-controlled withdrawal of CSF was carried out without removing the pressure transducer, via a tap connected to the pressure lines. This allowed us to assess CSFp while continuing to measure and record SSp during and after the end of CSF removal. Withdrawal was stopped when the pressure reached ~ 10 mmHg or if the patient started complaining of headaches and/or blurred vision.

### Direct retrograde cerebral venography [12–14]

The catheter inserted for pressure measurement with DRCV was longer and narrower than the manometry lines used for CSF pressure.

SSp was monitored and recorded with ICM+ in the same way as CSFp. The mean pressure level, slow vasogenic waves (period from 20 s to 2 min), and amplitude of pulse waveform (AMP_SSp_) were extracted through computer data analysis and recorded alongside CSFp.

### Statistical analysis

Data points of all parameters were distributed normally and hence the paired Student *t* test comparison was used for assessing the significance of any differences in pressures. A simple linear regression model was used to assess any associations between pairs of data.

### Ethics statement

All tests were performed as part of routine clinical management. All patients consented to the use of their data recordings for research purposes. At the time of this study, such consent did not include permission for data sharing.

## Results

**Fig. 1:**
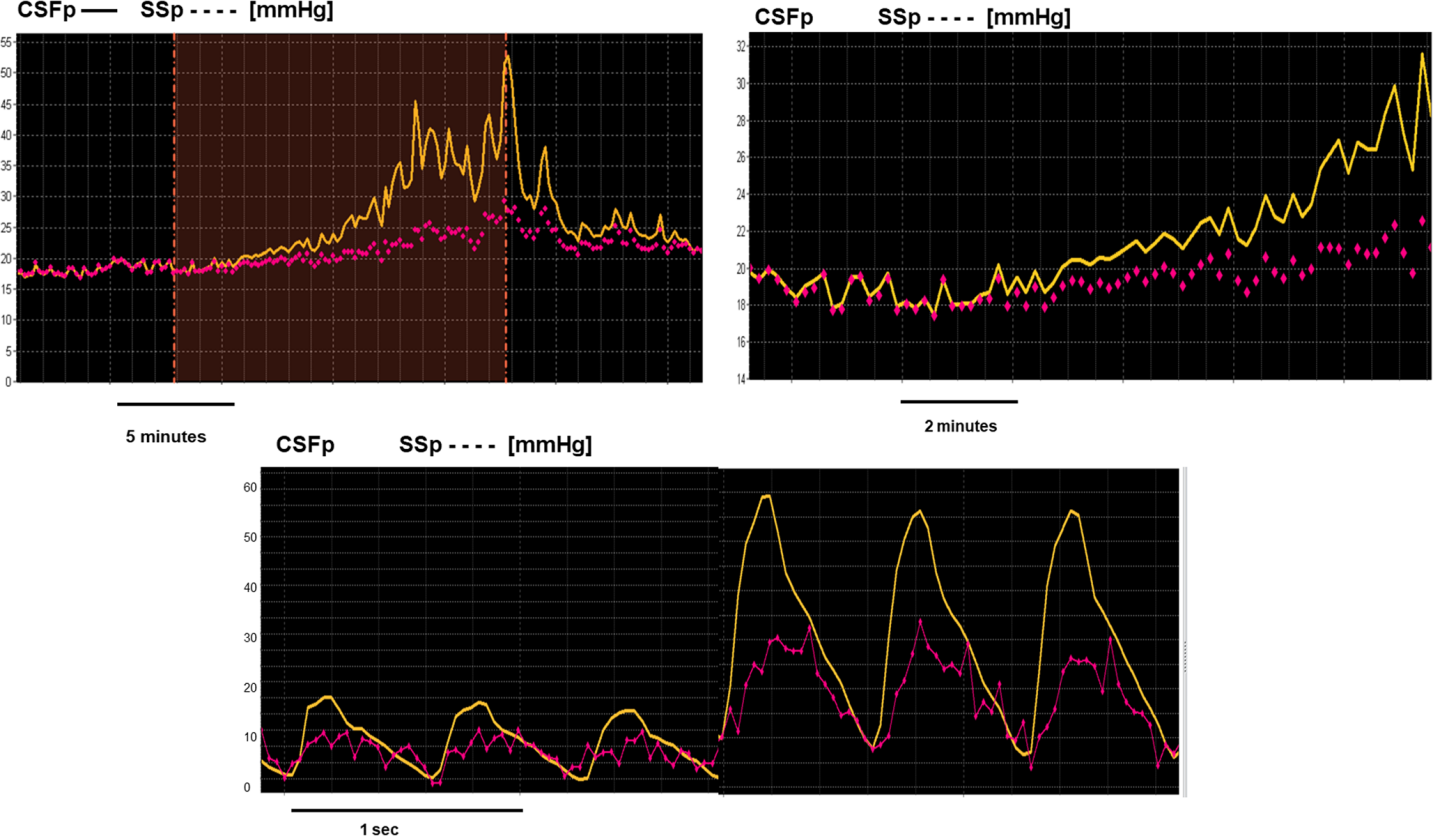
Observational demonstration of the static and dynamic coupling between CSFp and SSp. **a** Static coupling between the mean CSFp (upper, darker trend) and mean SSp (lower, dotted trend) values at baseline, during and after the end of infusion (the infusion period is marked as an event represented by the white area in the graph). **b** Dynamic coupling between the slow vasogenic waves of CSFp (upper, darker trend) and SSp (lower, dotted trend). **c** Coupling between the pulse amplitudes of CSFp and SSp at baseline and during infusion. CSFp, cerebrospinal fluid pressure; SSp, pressure of the sagittal sinus

Overall, we observed time-related coupling between mean CSFp and SSp **(**Fig. 1a), slow waves of CSFp and SSp **(**Fig. 1b), and pulse waveforms of CSFp and SSp. Pulse waveforms increased during an increase in both pressures provoked by infusion. Specifically, in cases when SSp amplitude was detectable (it was not possible to record any amplitude of SSp in 3 out of 10 sessions), both waveforms were adjacent in their diastolic phases and divergent during systole **(**Fig. 1c).

**Fig. 2:**
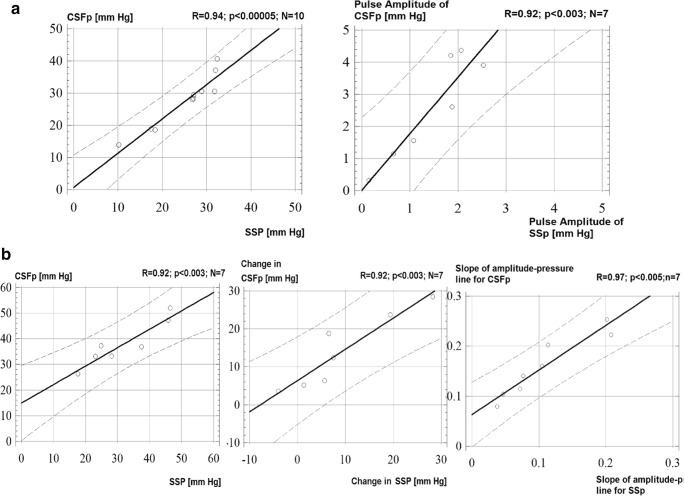
Coupling of CSFp and SSp at baseline and during infusion. **a** Left: linear regression demonstrating the coupling between CSFp and SSp at baseline. Right: coupling between the pulse amplitude of CSFp and SSp at baseline. Recording of the amplitude was only possible in 7 out of the 10 cases. **b** Linear regression demonstrating the maintenance of the coupling between CSFp and SSp during infusion (left), the strong correlation of the changes between CSFp and SSp during infusion, when CSFp is increased by infusion of Hartmann’s solution, and the increase in CSFp is subsequently invoking parallel increases in SSp (middle). Right: correlation between the slopes of the amplitude-pressure regression lines of both CSFp and SSp

Mean SSp correlated very strongly with the CSFp at baseline: *R* = 0.96; *p* = 0.0001; *N* = 9. Also at baseline, the pulse amplitudes of CSFp and SSp were well correlated (amplitude of SSp at baseline was recorded in 7 cases) (Fig. 2a).

During infusion, the two pressures increased concomitantly (*R* = 0.92; *p* < 0.003; *N* = 7) and the changes of both pressures correlated strongly (*R* = 0.97; *p* = 0.0007; *N* = 6). The slopes of the amplitude—pressure lines, calculated from a simple, linear regression model between CSFp and AMP of CSFp, and SSp and AMP of SSp—also correlated strongly during infusion (*R* = 0.97; *p* < 0.005; *N* = 7) (Fig. 2b).

Table 1 summarises the mean values and difference between CSFp and SSp at each phase of monitoring.

**Table 1:** Mean values of pressures during baseline, infusion and drainage

	CSF pressure (mmHg)	Sinus pressure (mmHg)	*p* value	CSFp-SSp (mmHg)	*p* value
Baseline	27.0 ± 2.3	25.2 ± 7.5	*p* = 0.026; *N* = 10	2.34 ± 2.72	0.01953
Infusion	38.0 ± 8.0	33.1 ± 12.0	*p* = 0.01; *N* = 7*	4.9 ± 4.0	*p* = 0.026; *N* = 7*
Drainage	12.7 ± 5.6	16.0 ± 2.7	*p* = 0.02; *N* = 8	− 3.2 ± 3.9	*p* = 0.0097; *N* = 8

*In 3 patients, only drainage was performed, as baseline CSFp was > 40 mmHg

*CSFp* cerebrospinal fluid pressure, *SSp* pressure of the sagittal sinus

**Fig. 3:**
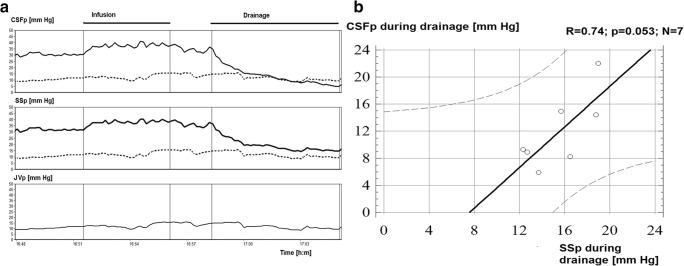
Correlation between CSFp and SSp during CSF drainage. **a** Overview of CSFp and SSp and JVP during infusion and during drainage of CSF. JVP is projected as a dotted line on the CSFp and SSp (SSp) panels, demonstrating that CSFp continues to drop after reaching JVP; in contrast, SSp reaches values close to JVP (CVP) and remains stable at this value as CSFp continues to decrease. **b** Correlation between CSFp and SSp during drainage of CSF. JVP, jugular venous pressure

The jugular venous pressure was measured in 5 patients and on average was 10.43 ± 3.8 mmHg. The jugular venous pressure (JVP) during one of the infusion tests is shown in Fig. 3a. Central venous pressure was measured on one patient and was relatively stable during infusion (11.6 ± 2.2 mmHg).

During drainage, the overall correlation between the 2 pressures was *R* = 0.78; *p* = 0.065, *N* = 6 (Fig. 3b**)**. During drainage, SSp appeared to stabilise at a level close to jugular vein pressure while CSFp continued to fall.

Table 2 summarises the differences between CSFp, SSp, and JVP at the end of drainage.

**Table 2:** Differences between CSFp, SSp, and JVP at the end of drainage in *N* = 5 patients with JVP measured

	Difference (mmHg)	Significance of difference
CSFp-JVP (mmHg)	− 2.2 ± 3.4	*p* = 0.026
SSp-JVP (mmHg)	4.27 ± 3.0	*p* = 0.004

### SSp as a function of CSFp

**Fig. 4:**
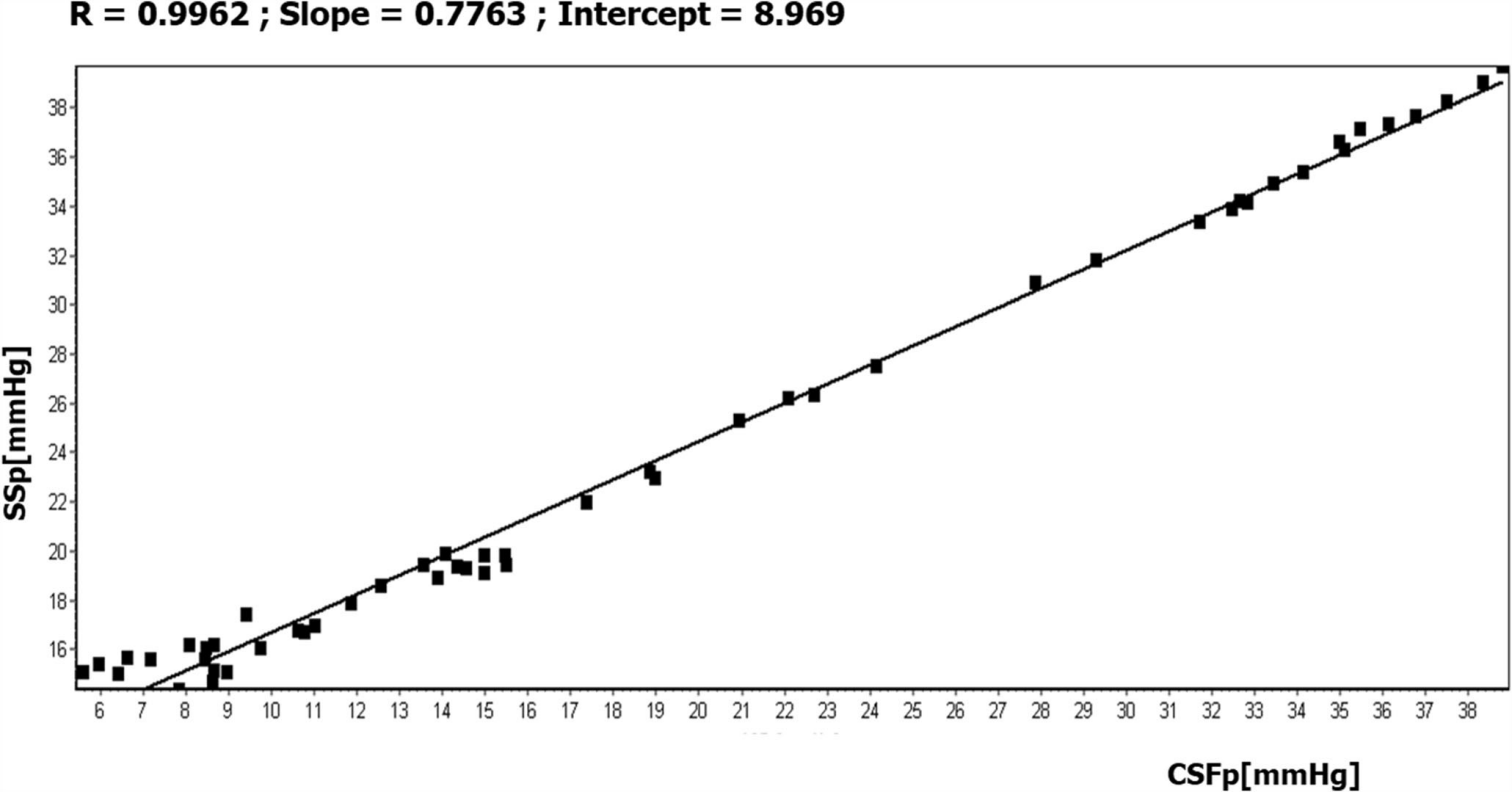
Example of the linear regression analysis between CSFp (ICP in the figure) and SSp (venous in the figure) for 1 out of the 9 studied patients. The slope and the intercept of the regression for each patient were averaged and were used to express SSp as SSp = *a* × CSFp + *b*, *a* = slope and *b* = intercept. SSp, expressed this way, can be used in Davson’s equation to simplify the calculations of its parameters in IIH patients. Notice that below CSFp 10 mmHg, SSp stopped to decrease further, while CSFp was easily drawn down by further drainage (JVP was 8 mmHg in this patient, CVP was ~ 11 mmHg)

Using linear regression for each individual patient, SSp was expressed as a function of CSFp in the format SSp = *a* × CSFp + *b* (for example, Fig. 4).

In all patients who had undergone infusion and drainage, ‘a’ was calculated to be 0.70 ± 0.14 for 9 patients. ‘b’ was calculated as 6.3 ± 3.5 mmHg which represents the intercept of this correlation that physiologically should correspond to central venous pressure (CVP). In the one patient, CVP was measured and found to be 11 mmHg; the intercept of the correlation was 9.2 mmHg, which is within the limits of measurement error (e.g. zeroing of external transducers).

## Discussion

Our results indicate the following, in many cases of PTCS:

- CSFp and SSp are coupled both statically (mean values) and dynamically (vasogenic components, mainly slow waves of CSFp and respiratory amplitude in CSFp and SSp).
- When CSFp increases during CSF infusion, it produces an increase in SSp and its vasogenic components.
- During drainage, both pressures decrease until a certain point (most probably JVP) when CSFp may decrease further while SSp remains constant.

Thus, venous sinus narrowing in PTCS generates significantly raised CSFp in contrast to healthy normal subjects [20, 22, 24]. There are other conditions in which SSp may not remain constant during CSF infusion as in infants and in the presence of an open fontanelle, myelomeningocoele, or Chiari malformation, and in individual cases [1, 5, , 27, 28, 31]. On the other hand, cerebral venous thrombosis and narrowing of the cerebral venous sinuses secondary, for example, to an intrasinus meningioma are recognised causes of PTCS where SSp is elevated but unchanged during CSF infusion.

### Coupling of the two pressures

After the start of infusion, even though there is a direct coupling between changes in CSFp and SSp, the two pressures appeared to diverge compared with baseline (as shown in Fig. 1). This divergence between mean CSFp and SSp may reflect that, at the beginning of the CSF infusion, all the infused fluid is initially accommodated within the intracranial compliant space. As CSFp increases towards its plateau, the infused CSF is absorbed into the sagittal and transverse sinuses [2, 3, 6].

### Implications of coupling between CSFp and SSp for the calculation of CSF outflow resistance

Davson’s equation refers to the steady state and assumes that SSp is independent of CSFp:

$$ \mathrm{CSFp}=\mathrm{Rout}\times {\mathrm{I}}_{\mathrm{f}}+\mathrm{SSp} $$

where Rout is the resistance to CSF outflow and *I*_f_ is the CSF formation rate [6].

In hydrocephalus, when SSp is not coupled to CSFp, a constant-rate infusion study enables the calculation of Rout as:

$$ \mathrm{Rout}=\left({\mathrm{CSPp}}_{\mathrm{plateau}}-{\mathrm{CSFp}}_{\mathrm{baseline}}\right)/\mathrm{Infusion}\ \mathrm{rate} $$

In the ten patients with PTCS in this study, the Rout calculated without correction for changes in SSp is 16.1 ± 2.1 mmHg/(ml/min). Using the measured values of SSp, Rout is calculated as 5.2 ± 1.4 mmHg/(ml/min) (*p* < 0.001), approximately 67% lower.

One approach is to modify Davson’s equation by expressing SSp as a function of CSFp (Fig. 4: SSp = a × CSFp + b). Therefore, Davson’s equation may be rewritten for PTCS as:

$$ \mathrm{CSFp}=\mathrm{Rout}\times {\mathrm{I}}_{\mathrm{f}}+\mathrm{a}\times \mathrm{CSFp}+\mathrm{b} $$

and subsequently,

$$ \mathrm{CSFp}=\left(\mathrm{Rout}\times {\mathrm{I}}_{\mathrm{f}}+\mathrm{b}\right)/\left(1-\mathrm{a}\right) $$

The average product of Rout × I_f_ is 3 mmHg [8], 1.5 in IIH with the corrected Rout (see above); therefore, the average CSFp = (1.5 + 6.3)/0.3 = 26 mmHg.

### Pathophysiological interpretation of Davson’s equation in PTCS

Finally, as derived from the simplified Davson’s equation for IIH, CSFp is increased and is estimated to be around 26 mmHg. This estimation is almost identical to the mean baseline CSFp in our patients, which is calculated as 27 mmHg. The derived formula explains why in IIH with CSFp-SSp coupling, the baseline intracranial pressure is elevated. Moreover, correcting Rout as derived by Davson’s equation, gives a more realistic estimation of a Rout on average < 7 mmHg × min/ml. We have observed in most of our classic IIH patients, that CSFp at plateau is generally not much higher than baseline, resembling the normal CSF circulation, as opposed to hydrocephalus patients. In few exceptional cases, where a higher than expected CSF plateau is observed, knowledge of the SSp could provide valuable information about the differential diagnosis.

Malm and colleagues [23], using a constant pressure infusion technique, demonstrated that there may be two groups of PTCS patients—one group with genuinely reduced conductance (increased Rout) and a second group with increased SSp as the cause of their impaired CSF absorption. They also showed that changes in CSF conductance changed with time after onset of PTCS.

However, these interpretations do not take into account the spatially distributed nature of CSF absorption. In most situations, CSF absorption is probably mainly intracranial and there is only a small gradient of pressure between the sagittal sinus and jugular foramen so that it is reasonable to use one single value for SSp. In contrast, in PTCS, there may be two CSF absorption pressure gradients—above and below an area of sinus narrowing. In other words, one Davson’s equation is required to describe CSF absorption upstream of the stenosis and another Davson’s equation for downstream absorption. Davson’s equation assumes that all the infused CSF is drained through channel(s) that may be described by a single parameter. If CSF absorption is split between upstream and downstream channels, Davson’s equation cannot be used as the relative proportion of ‘*I*_f_’ drained by the two systems is unknown. The transverse sinus pressure/JVP below the level of the stenosis is much lower than SSp above the stenosis. Lublinsky and colleagues have recently demonstrated the presence of arachnoid granulations in the transverse sinuses in both normal subjects and patients with IIH [21]. Interestingly, the total volume and interface contact area of intracranial arachnoid granulations is increased in IIH patients.

The situation may become even more complex if the stenosis is reversible with CSF removal and behaves as a Starling resistor [29]. If part of the transverse sinus is compressible, any rise in CSFp can decrease its lumen and increase the hydrodynamic resistance for sinus blood flow, increasing in the same way the SSp (if cerebral blood flow stays constant), which in turns increases CSFp. This mechanism works as a ‘vicious circle’ until CSFp and SSp reach an elevated state of equilibrium. This has been previously numerically simulated using an elegant mathematical model. The model forecasted that the system with collapsible transverse sinus (represented as a ‘Starling Resistor’) has two steady states: at low and at high CSFp.

### CSFp-SSp coupling in other intracranial pathologies

An important question that merits systematic study is whether the phenomenon of direct coupling of CSFp to SSp is limited only to PTCS or may also play a role in some cases of acute intracranial hypertension seen during brain swelling (head injury, stroke, meningitis, etc.). A study in this direction from early work suggested that 60% of ICP should be attributed to vascular mechanisms, rather than CSF circulatory component [18–20, 22, 32]. In one post-TBI patient, we anecdotally studied with double SSp and CSFp measurement during an infusion study; SSp appeared to stay constant when CSFp elevated.

Finally, our statistically strong and significant findings could have important implications for PTCS patients, both in the adult and paediatric populations, and it would be worth designing future randomised trials aiming at treating PTCS patients by stopping the reported pathophysiological coupling of the two pressures.

## Limitations

We did not collect information about arterial blood pressure during the infusion studies and therefore its role in the interaction between the CSF and arterial/venous blood flows for these PTCS patients.

Unfortunately, full analysis of the frequencies of the CSFp and SSp/JVP is not possible using retrospective data because we did not have information about the frequency properties of the two pressure measurement systems used: in CSFp, a short and wide manometer line and a LP needle was used; however, in SSP, a long thin catheter and external transducer. This makes accurate spectral analysis on CSFp-SSp questionable.

## Conclusion

CSFp and SSp are coupled in PTCS, both at baseline and during infusion, forming a positive feedback loop that may be interrupted by CSF drainage. The implications of the calculation of CSF outflow resistance are discussed.

## Data Availability

Unfortunately, we do not possess appropriate ethics in order to share our anonymised data from patients, as all studies were performed on a clinical indication and at the time of consent there was no statement asking permission for sharing.
